# Structure and magnetic properties of W-type hexaferrites

**DOI:** 10.1107/S2052252519003130

**Published:** 2019-04-30

**Authors:** Mathias I. Mørch, Jakob V. Ahlburg, Matilde Saura-Múzquiz, Anna Z. Eikeland, Mogens Christensen

**Affiliations:** a Center for Materials Crystallography, Department of Chemistry and Interdisciplinary Nanoscience Center (iNANO), Aarhus University, Langelandsgade 140, Aarhus C 8000, Denmark

**Keywords:** hexaferrites, magnetic structures, combined neutron and X-ray refinement, W-type hexaferrites

## Abstract

W-type hexaferrites (WHFs) (Sr*Me*_2_Fe_16_O_27_, *Me* = Mg, Co, Ni and Zn) are hard magnetic materials with high potential for permanent magnet applications owing to their large crystalline anisotropy and high cation tunability. However, little is known with regards to their complex structural and magnetic characteristics. Here, the substitution of metals (*Me* = Mg, Co, Ni and Zn) in WHFs is described and their crystal and magnetic structures investigated. From joined refinements of X-ray and neutron powder diffraction data, the atomic positions of the *Me* atoms were extracted along with the magnetic dipolar moment of the individual sites. The four types of WHFs exhibit ferrimagnetic ordering. For Mg, Ni and Zn the magnetic moments are found to be ordered colinearly and with the magnetic easy axis along the crystallographic *c* axis. In SrCo_2_Fe_16_O_27_, however, the spontaneous magnetization changes from uniaxial to planar, with the moments aligning in the crystallographic *ab* plane. Macromagnetic properties were measured using a vibration sample magnetometer. The measured saturation magnetization (*M*_s_) between the different samples follows the same trend as the calculated *M*_s_ extracted from the refined magnetic moments of the neutron powder diffraction data. Given the correlation between the calculated *M*_s_ and the refined substitution degree of the different *Me* in specific crystallographic sites, the agreement between the measured and calculated *M*_s_ values consolidates the robustness of the structural and magnetic Rietveld model.

## Abstract 2

W-type hexaferrites were investigated by both X-ray and neutron powder diffraction along with macroscopic measurements. The refined nuclear structure and magnetic ordering are in good agreement with the measured magnetic properties, solidifying the robustness of using X-rays and neutrons in conjunction.

## 1. Introduction   

Permanent magnetic materials are a keystone in society today (Jacobs, 1969 ▸). Magnets have enabled the information age by providing data storage (Coey, 2001 ▸) and allowed generation of electricity through induction (Lewis & Jiménez-Villacorta, 2013 ▸). Given their importance in the modern world, there is a big incentive to improve magnets and the most common figure of merit for magnetic materials is the energy product (*BH*_max_) (McCallum *et al.*, 2014 ▸), *i.e.* the largest rectangle in the second quadrant under the *BH* curve in the hysteresis loop (O’Handley, 2000 ▸). Nd_2_Fe_14_B magnets have been the best performing magnets since they were discovered in 1982 and have continuously improved through laborious developments (Brown *et al.*, 2002 ▸). Tightened export quotas of rare-earth elements (REE) in late 2009 (Bomgardner, 2015 ▸; Wübbeke, 2013 ▸), along with health concerns working with REE ores (International Atomic Energy Agency, 2011 ▸; Pagano *et al.* 2015 ▸) have prompted research of REE-free magnets. A widespread REE-free permanent magnet is the M-type hexaferrite (MHF), *e.g.* BaFe_12_O_19_ or SrFe_12_O_19_ (Pullar, 2012 ▸). Although not as powerful as Nd_2_Fe_14_B magnets, ferrites are the most abundantly used permanent magnetic material throughout the world as a result of their low cost, high stability and adequate performance in many applications (Pullar, 2012 ▸; Lewis & Jiménez-Villacorta, 2013 ▸). Recent work has focused on optimizing the performance of MHFs through bottom-up nanostructuring and morphology control (Eikeland *et al.*, 2018 ▸, 2017 ▸; Saura-Muzquiz *et al.*, 2016 ▸, 2018 ▸). However, there is still a huge gap in performance between REE magnets and REE-free magnets (Coey, 2012 ▸), encouraging further research into REE-free magnets. Although the MHF is the best known and widely commercially used ferrite, several other hexaferrite structures exist of which an overview can be found in the review by Pullar (2012 ▸). One of the promising candidates is the W-Type hexaferrite (WHF): *A* ^2+^ *Me* ^2+^Fe_16_^3+^O_27_, (*A* = alkaline earth metal, *e.g.* Sr or Ba, *Me* = Mg, Mn, Fe, Co, Ni, Cu, Zn). These hexaferrites hold great potential as future permanent magnetic materials owing to the wide tuneability of their magnetic properties.

**Figure 1:**
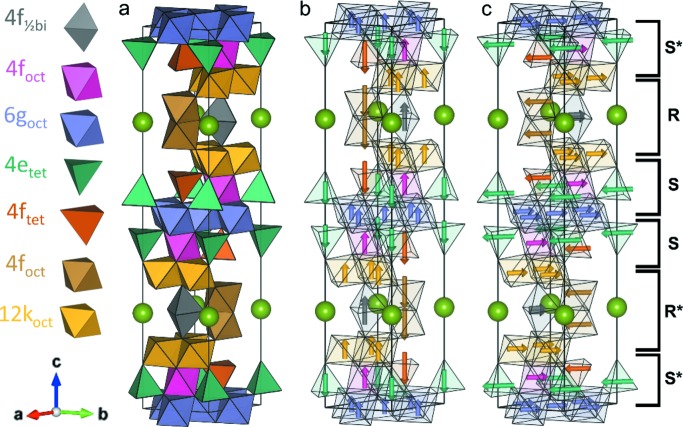
(*a*) Unit cell and seven distinct *Me* sites for Sr*Me*_2_Fe_16_O_27_ (*Me* = Mg, Co, Ni, Zn), the green spheres are Sr and the colored polyhedra show the *Me* sites and their relative coordination. Oxygen atoms at the corners of polyhedra are not shown. (*b*) Uniaxial magnetic ordering of SrMg_2_Fe_16_O_27_, SrNi_2_Fe_16_O_27_ and SrZn_2_Fe_16_O_27_. (*c*) Planar magnetic ordering of SrCo_2_Fe_16_O_27_. The brackets in the right part of the figure indicates the common hexaferrite layers. Figure prepared using *VESTA* (Momma & Izumi, 2011 ▸).

In the common descriptions of hexagonal ferrites (Pullar, 2012 ▸; Smit & Wijn, 1959 ▸), R denotes a layer including an *A* ion, whereas S describes a layer with a spinel-like structure, and an asterisk (*) next to R or S denotes layers that are rotated 180° around the crystallographic *c* axis. According to this notation, the MHF can be described as RSR*S*, whereas WHFs can be described by introducing an additional S layer into the MHF structure, resulting in the structure RSSR*S*S*. Fig. 1 ▸ shows the WHF structure along with the obtained magnetic structures from neutron powder diffraction investigations obtained in this work. The introduction of the additional spinel layer in the WHF structure gives rise to a 6*g*_oct_ site which lies on the SS boundary. In the most conventional spin-arrangement (Gorter, 1957 ▸), the ratio of sites with parallel/antiparallel spin alignment per unit cell is maintained. WHF (12↑/6↓) compared with MHF (8↑/4↓) has a 50% net increase in parallel magnetic sites whereas the unit cell volume only increases by roughly 42–44% depending on *Me* substitution. Introduction of the additional S layer necessitates two *Me* ^2+^ ions in the structure, as in spinel-ferrites, which enable the direct substitution of magnetic or nonmagnetic *Me* ^2+^ ions without the need for additional substitutions to maintain the charge balance (Tokunaga *et al.*, 2010 ▸; Wang *et al.*, 2012 ▸). This enhances the tuneability of WHF in comparison with MHF (Gorter, 1950 ▸; Andersen *et al.*, 2018 ▸). By partially substituting nonmagnetic species into specific sites, which align opposite to the net magnetization of the unit cell, *i.e.* the tetrahedrally coordinated 4*e* and 4*f* sites in the S layer and the octahedral 4*f* site in the R layer, the magnetization of the compound can be potentially increased with minimal reduction of the super-exchange coupling in and between the layers (Lilot *et al.*, 1982 ▸; Ram & Joubert, 1991*b* ▸).

Ease of synthesis has caused research to primarily focus on Ba-containing WHFs (Collomb *et al.*, 1986 ▸ *a*, ▸ *c*, 1988 ▸; Lotgering *et al.*, 1980 ▸ *b*; Ahmed *et al.*, 2009 ▸; Cao *et al.*, 2018 ▸), but considering Ba health concerns (Choudhury & Cary, 2001 ▸), further research towards Sr-containing WHFs is highly interesting as they are nontoxic (Kirrane *et al.*, 2006 ▸). Some efforts have already gone into Sr-containing WHFs: Ram & Joubert (1991*a* ▸) reached a saturation magnetization (*M*_s_) of 91 A m^2^ kg^−1^ at room temperature by Li/Zn substitution (SrZnLi_0.5_Fe_16.5_O_27_). This value is about 25% larger than achieved for MHFs. Toyota (1997 ▸) also reported a *BH*_max_ of 42 kJ m^−3^ for SrFe_18_O_27_, which is significantly higher than for pure SrFe_12_O_19_, normally limited to about 30 kJ m^−3^ (Eclipse Magnetics Ltd, 2019 ▸; Eikeland *et al.*, 2017 ▸). Furthermore, WHFs have also revealed themselves as promising materials for other applications, *e.g.* multiferroics (Song *et al.*, 2014 ▸; Kimura, 2012 ▸) or microwave absorbance (Ahmad *et al.*, 2012 ▸). As Sr-containing hexaferrites in general do not always display the same cation distribution as Ba-containing hexaferrites (Albanese *et al.*, 1973 ▸; Sizov, 1968 ▸), it is highly relevant and interesting to investigate both the cation distribution and subsequent magnetic ordering of the Sr-containing hexaferrites. Aside from the cation distribution, the crystallite size also has a huge impact on the macroscopic magnetic properties of hexaferrites (Gjørup *et al.*, 2018 ▸) and is an important part of optimizing their performances.

This work involved rigorous investigation of Sr*Me* ^2+^Fe_16_^3+^O_27_ (where *Me* = Mg, Co, Ni and Zn) synthesized by high-temperature sol-gel synthesis. Neutron powder diffraction (NPD) data along with X-ray powder diffraction (PXRD) data extracted using both synchrotron and laboratory sources were analyzed with combined Rietveld refinements. The data obtained were used to determine the magnetic structure and the occupation of transition metal atoms in the hexaferrite structures. The crystallographic structural and magnetic data is compared with macroscopic magnetic measurements.

## 2. Experimental   

### 2.1. Synthesis   

Sr*Me*_2_Fe_16_O_27_ particles were synthesized utilizing a sol-gel autocombustion method due to the high chemical homogeneity achieved in these syntheses (Hench & West, 1990 ▸). The method contains three steps: first Sr(NO_3_)_2_, *Me*(NO_3_)_2_·6H_2_O and Fe(NO_3_)_3_·9H_2_O (all Sigma-Aldrich technical grade with purity >98%) were dissolved in demineralized water in stoichiometric molar ratios [Sr^2+^]:[*Me* ^2+^]:[Fe^3+^] = 1:2:16. Citric Acid was dissolved and added in equal ratio to the nitrates, [C_8_H_8_O_7_] = 2[Sr^2+^] + 4[*Me* ^2+^] + 48[Fe^3+^], under constant stirring. The solution was neutralized with NH_4_OH and dried overnight in a convection oven at 100°C until a gel was formed. In the second step, the gel was fired in a preheated furnace at 350°C for 30 min until the autocombustion had finished and subsequently cooled to room temperature in air. Finally, the resulting powder was crushed and fired in a furnace at 1200°C (SrMg_2_Fe_16_O_27_ and SrZn_2_Fe_16_O_27_) or 1300°C (SrNi_2_Fe_16_O_27_ and SrCo_2_Fe_16_O_27_) according to the following heating scheme: 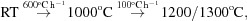
with a holding time of 2 h before cooling to room temperature.

### 2.2. Powder diffraction   

Four diffraction data sets were measured for all of the prepared WHF samples and were collected using the following instruments.

*X-rays*: (i) Synchrotron radiation at the MS beamline at SLS, PSI, Switzerland (Willmott *et al.*, 2013 ▸) with a chosen wavelength of λ = 0.778 Å. The samples were packed in 0.3 mm capillaries and the data were collected using Debye–Scherrer geometry and a Mythen-II detector in the angular range 2θ = 2–121°. (ii) An in-house Rigaku SmartLab diffractometer equipped with a Co *K*α source (*K*α_1_ = 1.789 and *K*α_2_ = 1.793 Å). Data were collected in parallel-beam geometry in reflection mode in the angular range 2θ = 18–90° using a D/teX Ultra detector. The powder samples were packed on zero-background single-crystal silicon sample holders.

*Neutrons*: HRPT at SINQ, PSI, Switzerland (Fischer *et al.*, 2000 ▸) with chosen wavelengths (iii) λ = 1.89 Å and (iv) λ = 2.45 Å. The powders were packed in vanadium cans (ø = 6 mm) and data were collected in Debye–Scherrer geometry with a large position-sensitive ^3^He detector having an angular range of 2θ = 5–165°. During the measurement, the detector was shifted by 0.05° to enhance the number of data points across the diffraction peaks.

**Figure 2:**
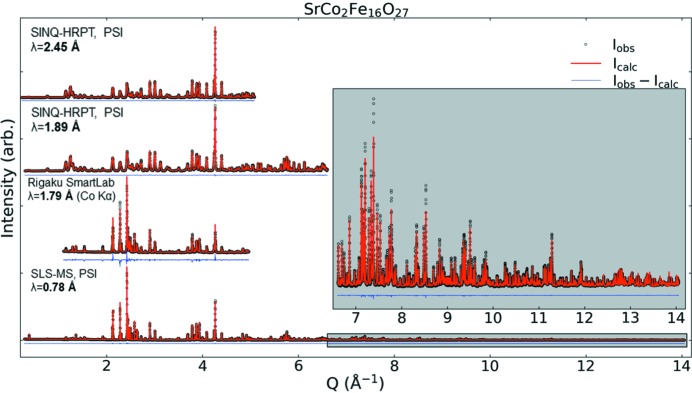
Four diffraction patterns and corresponding Rietveld refinements and difference spectra for SrCo_2_Fe_16_O_27_. Inset is data from SLS-MS at PSI for *Q* = 6.6–14 Å^−1^.

The four diffraction patterns were collected on the basis of their individual strengths, which improve the robustness of the analysis. The individual strengths attributed to each method are as follows. *X-rays*: (i) MS-SLS: high peak resolution and large coverage of *Q*-Space; (ii) Rigaku SmartLab: precise wavelengths (*K*α_1_ and *K*α_2_) for accurate *d* spacings and readily available for purity control, slight contrast effect due to Fe resonance *f*′_Fe_(λ). *Neutrons*: (iii) HRPT (1.89 Å): largest *Q*-space coverage of neutrons; (iv) HRPT (2.45 Å): highest resolution at low *Q* of neutron powder diffraction data. All Rietveld refinements were carried out using the *FullProf Suite* software package (Rodríguez-Carvajal, 1993 ▸), where four combined refinements of four sets of diffraction patterns were performed. Fig. 2 ▸ shows the four different patterns with accompanying Rietveld refinements and difference plots for SrCo_2_Fe_16_O_27_. The refinements of the other WHFs are shown in Figs. S1–S3 of the supporting information.

#### 2.2.1. Technical refinement details   

All four samples belong to the space group *P*6_3_/*mmc,* unit-cell parameters and isotropic thermal parameters are given in Table S4 of the supporting information. The peak profile was described with the Thomson–Cox–Hastings pseudo-Voigt profile with axial divergence asymmetry (Thompson *et al.*, 1987 ▸), where *Y* and *X* are the only profile parameters refined relating to size and strain, respectively. Instrumental broadening effects were described by refinement of standards at the MS beamline [Si NIST 640D) with a Rigaku SmartLab diffractometer (LaB_6_ NIST 660B)] and at HRPT (Na_2_Al_12_Ca_3_F_14_); all standards were measured under the same conditions as the samples. Figs. S10 and S11 show the pseudo-Voigt FWHM of the different instruments as a function of *Q* and 2θ. Peak-broadening resulting from the samples was constrained to be equal between the different patterns with *Y* constrained with respect to the difference in wavelength (*X* has no wavelength dependency). For all patterns of a specific sample, the unit-cell parameters and atomic positions within the unit cells were constrained to be identical, as they should not vary across the different measurements carried out under equal conditions. A total of three isotropic thermal vibration parameters were used, describing Sr, Fe/*Me* and O. Fourier cosine series were used to model the neutron background in HRPT patterns, whereas Chebychev polynomials were used for the X-ray patterns. The refined magnetic moments between Fe^3+^, Co^2+^ and Ni^2+^ were constrained to maintain the ratio between the number of unpaired 3*d* electrons of the individual elements, assuming the orbit moment to be quenched, *e.g. μ*_B_(Fe^3+^):μ_B_(Co^2+^) = 5:3. The difference in neutron scattering length of Fe (9.45 fm) compared with Co (2.49 fm), Ni (10.6 fm) and Zn (5.68 fm) made it possible to determine the site occupancies of the neighboring *Me* elements in the refinements. The initial predictions for the *Me* occupancies were found by refining the structures with only Fe and looking at their individual thermal parameters. The occupancies were subsequently refined by introducing *Me* atoms into the sites where thermal parameters mostly deviated from the only Fe refinement, while constraining thermal parameters to be equal between all metal sites. The total site occupation is fixed to have all sites fully occupied.

### 2.3. Magnetic measurements   

The magnetic hysteresis loops were measured using a physical properties measuring system (PPMS) from Quantum Design equipped with a vibrating sample magnetometer (VSM). Powder samples of 20–45 mg masses were packed in cylindrical powder capsules (height 2.7–3.0 mm, diameter 3.5 mm), and measurements were performed at 300 K with an applied field of ±3 T. The graphical (near) infinite slope method was used to correct the data for demagnetization, whereas the approach to saturation was used to extract *M*_s_ (O’Handley, 2000 ▸). Fig. 7 shows hysteresis loops for all four WHFs.

Curie temperatures (*T*_C_) were measured with thermogravimetry using an STA 449 F3 Jupiter from Netzsch. Pellets of 60–120 mg were measured with attached external magnets by ramping the temperature at a rate of 10 °C min^−1^ in an Ar flow of 50 ml min^−1^. The weight in % of the original mass is plotted against temperature in Fig. S4 and the extracted Curie temperatures are given in Fig. 4.

## 3. Results and discussion   

### 3.1. Structural model   

The results of the combined refinement of all diffraction patterns are shown for SrCo_2_Fe_16_O_27_ in Fig. 2 ▸, while the refined model and patterns for Sr*Me*_2_Fe_16_O_27_ (*Me* = Mg, Ni and Zn) are given in Figs. S1–S3.

### 3.2. Purity and occupation   

Refinements showed almost phase-pure samples, with small impurities of CoFe_2_O_4_ [5.27 (5) wt%], MgFe_2_O_4_ [8.24 (5) wt%], NiFe_2_O_4_ [7.08 (6) wt%] and ZnFe_2_O_4_ [5.28 (4) wt%] which were quantifiable by the high-quality data from SLS-MS. The SrMg_2_Fe_16_O_27_ sample additionally contained a small impurity of X-type hexaferrite Sr_2_Mg_2_Fe_28_O_46_ [8.77 (5) wt%]. A few undescribed peaks in this dataset stem from a small impurity that remains to be determined but is assumed to be very small based on the peak intensities. A selection of the impurity peaks are highlighted in Figs. S12–S16. The resulting structure of the WHF, with seven distinct *Me*–O polyhedra that make up the structure, is shown in Fig. 1 ▸(*a*)

**Figure 3:**
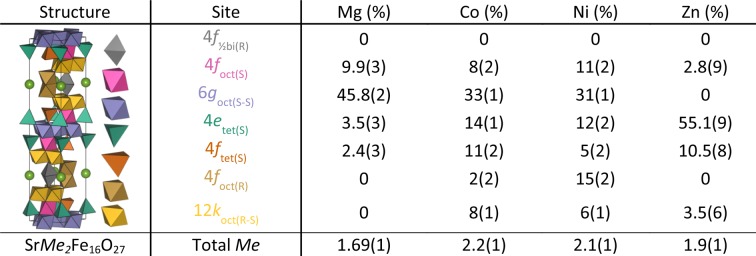
Overview of the occupation of *Me*s given as a percentage of the full site occupancies and the total amount of *Me* for one formula unit Sr*Me*_2_Fe_16_O_27_.

The refined occupation fractions (%) of the full site occupancies are given in Fig. 3 ▸. The sites occupied by *Me*s are as follows: in all samples the bipyramidal 2*d* site is displaced from the center and described by a half-occupied 4*f* site in the R-layer [4*f*_½bi(R)_], this is exclusively occupied by Fe. Mg predominantly occupies octahedral S sites [4*f*_oct(S)_ and 6*g*_oct(S-S)_] and, to a minor degree, tetrahedral S sites [4*e*_tet(S)_ and 4*f*_tet(S)_]. Zn predominantly occupies 4*e*_tet(S)_ and 4*f*_tet(S)_ with a minor occupation of 4*f*_oct(S)_ and 12*k*_oct(R-S)._ Co and Ni are spread across all sites, but the predominantly occupied site for both is 6*g*_oct(S-S)_. For SrNi_2_Fe_16_O_27_, the data does not support unambiguous determination of *Me* occupation, given the poor scattering length contrast between Ni and Fe for both neutrons and X-rays. Comparisons between the occupancies obtained in this work and in previously reported studies (Collomb *et al.*, 1986 ▸ *c*, ▸ *a*; Graetsch *et al.*, 1986 ▸) of (Ba/Sr)*Me*_2_Fe_16_O_27_ (*Me* = Mg, Zn and Co) are given in Tables S1–S3.

### 3.3. Magnetic structure   

From the NPD data, it is also possible to determine the magnetic structure as the spin of the neutrons interacts with the atomic magnetic dipole moment. Gorter (1957 ▸) proposed ferrimagnetic ordering in WHFs with the 4*e*_tet(S)_, 4*f*_tet(S)_ and 4*f*_oct(R)_ sites ordering antiparallel to the remaining sites and this has been demonstrated for BaCo_2_Fe_16_O_27_ using neutron scattering (Collomb *et al.*, 1986 ▸ *b*). The magnetic and crystallographic unit cells are coinciding and three possibilities for the orientation of the magnetic moments have been considered for the obtained data: (i) uniaxial ordering where the magnetic moments are ordered along the crystallographic *c* axis, (ii) planar ordering where the magnetic ordering is in the *ab* plane and (iii) conical ordering where the moments have an angle with respect to the *c* axis.

**Figure 4:**
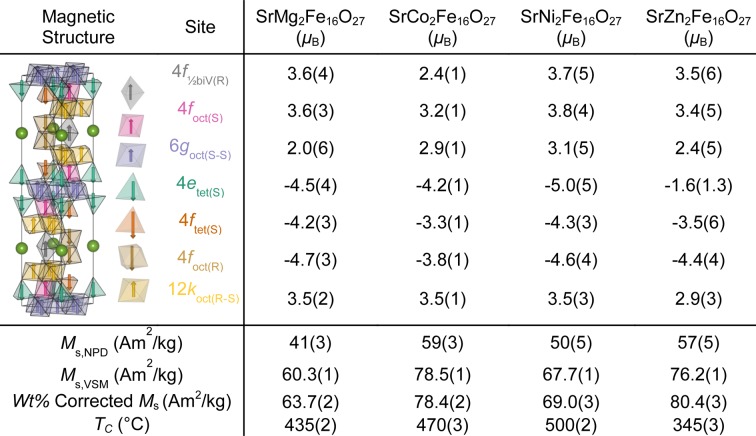
Average magnetic moments of the seven *Me* sites, the derived *M*_s_, the *M*_s_ from the macroscopic hysteresis measurement and the Curie temperature from thermogravimetry. Correction of *M*_s_ is with respect to impurity phases and is discussed in Section S8

**Figure 5:**
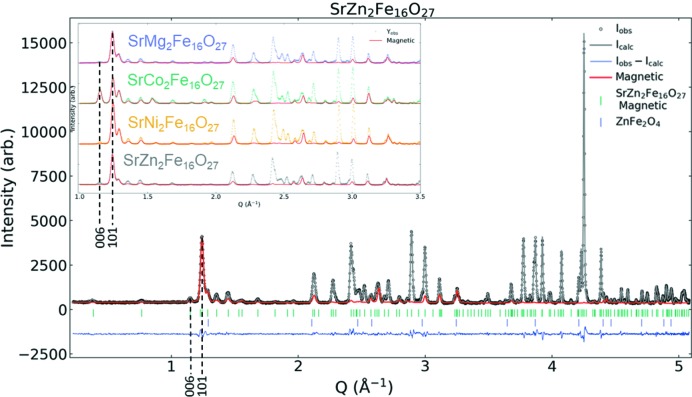
NPD data for SrZn_2_Fe_16_O_27_ measured at SINQ-HRPT λ = 2.45 Å. The contribution from the refined magnetic scattering is shown in red. The inset shows a comparison between the four different samples in the range *Q* = 1.0–3.5 Å^−1^ with the two dominant magnetic peaks indicated at 006 and 101 (see Fig. S9 for a magnified version). SrCo_2_Fe_16_O_27_ differs from the other three WHFs by having planar magnetic ordering instead of uniaxial ordering.

The propagation vector is zero (**k** = 0) as no additional magnetic peaks can be seen aside from those coinciding with the crystallographic Bragg peaks. Consequently, a collinear magnetic model is imposed with the same unit cell size as the crystallographic unit cell. For Mg, Ni and Zn there is no magnetic contribution to the (00*l*) reflections, indicating that the magnetic moment is aligned along the *c* axis. As the magnetic scattering is given by 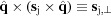 (Lefmann, 2017 ▸; Marshall & Lovesey, 1971 ▸), showing that the spin component on site j (**s**_j_) is only visible if it is perpendicular to the scattering vector 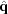. Refinement of the neutron diffraction data confirms that Sr*Me*_2_Fe_16_O_27_ (*Me* = Mg, Ni and Zn) can be satisfactorily described by a ferrimagnetic model, having the magnetic moments aligned along the *c* axis. The parallel and antiparallel alignment of magnetic moments on the different sites follows what is described by Gorter (1957 ▸) and as a result the magnetic structure belongs to the magnetic space group (Shubnikov group) *P*6_3_/*mm*′*c*′. The magnetic contribution to the diffraction signal is highlighted by a red line for SrZn_2_Fe_16_O_27_ and shown in Fig. 5, where the inset highlights the different magnetic signals for the respective WHFs. The refined magnetic moments from NPD were summed over all sites in the unit cell and divided by the mass of the unit cells as an estimation of *M*_s_. Fig. 4 ▸ shows the refined moments for each crystallographic site and the *M*_s_ calculated from *M*_s,NPD_, as well as the *M*_s_ extracted from the macroscopic magnetic measurement (*M*_s,VSM_) for comparison. In Figs. S5–S7, the refined magnetic contribution for all WHFs is shown in greater detail. A table of the refined positions of all seven *Me* sites along with the seven O sites is given in Table S5.

**Figure 6:**
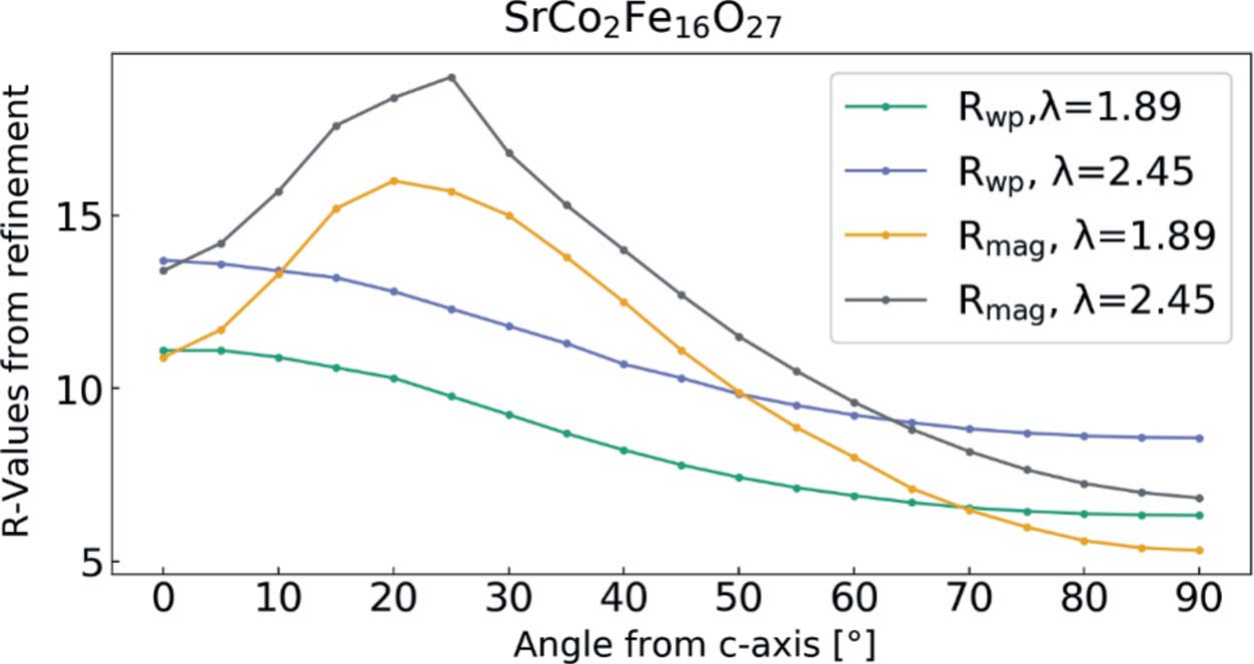
Resulting *R* factors from the refinements where the only difference is the constrained angle for the magnetic ordering with respect to the *c* axis. The *R* factors shown are for the refined model of the data measured at SINQ-HRPT at PSI.

While the PXRD is similar for the four different structures, there is a clear difference in the NPD in Fig. 5 ▸, the reason being that the magnetic structure of SrCo_2_Fe_16_O_27_ differs from the other WHFs. The strong peak at 1.15 Å^−1^ is the (006) peak. This peak dictates a component of the magnetic moment in the crystallographic *ab* plane, as magnetic scattering is perpendicular to the direction of the magnetic moment. Previous studies (Samaras *et al.*, 1989 ▸; Collomb *et al.*, 1986 ▸ *c*; Graetsch *et al.*, 1984 ▸; Lotgering *et al.*, 1961 ▸; Paoluzi *et al.*, 1988 ▸; Asti *et al.*, 1978 ▸) of BaCo_2_Fe_16_O_27_ have reported either a conical magnetic structure with an angle to the *c* axis of 69–71°, or planar magnetic ordering. To the best of our knowledge, the most accepted magnetic structure is the conical ordering reported by Samaras *et al.* (1989 ▸); however, the structure is known to be complex and changes as a function of both temperature and exact Co substitution, as described by Yamzin *et al.* (1966 ▸). To investigate the direction of the magnetic moment in SrCo_2_Fe_16_O_27_, the refinements were carried out with the orientation of the atomic magnetic dipolar moment varying in angle with respect to the *c* axis in steps of 5° from uniaxial (0° from the *c* axis) to planar (90° from the *c* axis). The magnetic ordering conical to, or in the *ab* plane defined by the magnetic space groups belonging under the crystallographic space group *P*6_3_/*mmc* have either 0 moment or are antiparallel within each Wyckoff site based on the magnetic space groups (Litvin, 2013 ▸). As a result, the symmetry restrictions on the magnetic moments were reduced, so each Wyckoff site must be equal and pointing in the same direction, but is not dictated by any magnetic group of *P*6_3_/*mmc*. In Section S5 of the supporting information, further discussion on the magnetic symmetry is given. A graph of the resulting *R* factors is shown in Fig. 6 ▸ while the decreases in *R* factors are small between conical and planar ordering, the lowest value is for 90° and there is no evidence to suggest that the ordering should be conical rather than planar. SrCo_2_Fe_16_O_27_ must belong to a lower symmetry space group than *P*6_3_/*mmc*, which remains to be determined.

Refinements were carried out with two options for peak broadening: (i) peak broadening of the magnetic contribution is constrained to be equal to that of the peak broadening of the nuclear contribution and (ii) the peak broadening for magnetic and nuclear contribution are independent. If the Lorentzian peak broadening attributed to size (Y) is refined independently, the refined broadening of the magnetic diffraction signal is an order of magnitude larger than the refined broadening of the nuclear contribution, along with a small decrease in *R*_wp_. A comparison of the peak width parameter Y along with the resulting *R*_wp_s is given in Table S7. The peak width originating from the crystal structure is very sharp suggesting the crystallite sizes to be out of resolution, on the other hand, the magnetic scattering has a size corresponding to ∼100 nm, which can be explained by the crystallites breaking up into smaller magnetic domains. The underlying property responsible for the difference in magnetic ordering between Sr*Me*_2_Fe_16_O_27_ (*Me* = Mg, Ni, Zn) compared with SrCo_2_Fe_16_O_27_ is a strong coupling of the unquenched orbital momentum present in Co and the trigonal axis of the crystal field, which has been described previously for cobalt-substituted magnetite (Slonczewski, 1961 ▸, 1958 ▸) and BaCo_2_Fe_16_O_27_ (Bickford, 1962 ▸). Samaras *et al.* (1989 ▸) have investigated the magnetic ordering for BaCo_2_Fe_16_O_27_ with an occupation of 70% Co^2+^ in the 6*g*_oct_ site, which exhibits the trigonal axis around it. They concluded that there is a tilt of the magnetic moment between 69 and 71°, *i.e.* conical ordering in contrast to the planar ordering (90°) observed here. A small difference in the total Co content [1.87 (7) Co^2+^ per formula unit (f.u.) *versus* 2.2 (1) Co^2+^ per f.u.] could possibly explain this discrepancy as the amount of Co has previously been shown to alter the tilt of the magnetic moments (Yamzin *et al.*, 1966 ▸).

In comparison with the theoretical work on superexchange interactions by Lilot *et al.* (1982 ▸), the *T*_C_ of the four samples can be related to how important each individual site is in the exchange interaction: 4*e*_tet_ > 4*f*_tet_ > [12*k*_oct_, 4*f*_oct(R)_] > [4*f*_½bi_, 6*g*_oct_, 4*f*_oct(S)_]. This is done by relating the cation substitution and magnetic moment on different crystallographic sites to the measured *T*_C_ of the samples. The two samples with diamagnetic substitutions have the lowest *T*_C_, leading to *T*_C_ values of ZnWHF < MgWHF < CoWHF < NiWHF. The low *T*_C_ seen for SrZn_2_Fe_16_O_27_ can be understood by the diamagnetic Zn^2+^ cation occupying the two most influential sites for the superexchange, 4*e*_tet_ and 4*f*_tet_. SrMg_2_Fe_16_O_27_ is the second lowest owing to the diamagnetic Mg^2+^. SrCo_2_Fe_16_O_27_ has a slightly higher Co occupation in 4*e*_tet_ and 4*f*_tet_ sites and additionally, the magnitude of the moment is lower on these important antiparallel sites (see Fig. 4 ▸) when compared with SrNi_2_Fe_16_O_27_, which has the highest *T*_C_ of these four samples. The Curie temperatures here are in agreement with previously reported values for Ba*Me*_2_Fe_16_O_27_, where *Me* = Co, Mg (Collomb *et al.*, 1986 ▸ *a*) and *Me* = Ni, Zn, Cu (Besagni *et al.*, 1981 ▸; Licci *et al.*, 1981 ▸).

### 3.4. Magnetic measurements   

**Figure 7:**
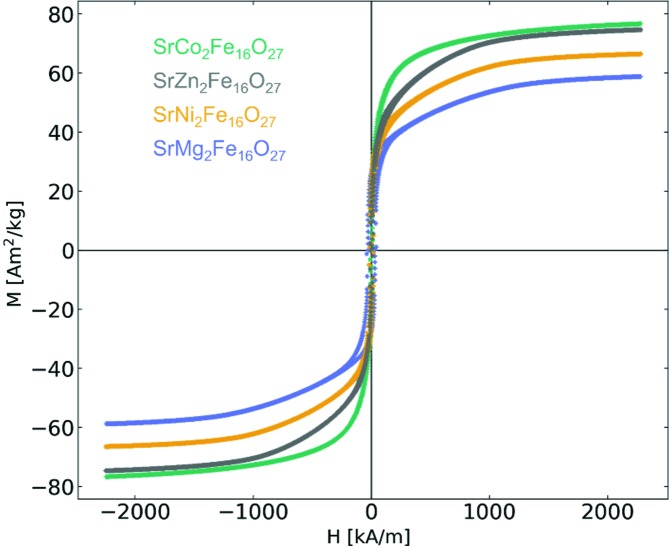
Magnetic hysteresis for the four Sr*Me*_2_Fe_16_O_27_ (*Me* = Mg, Co, Ni, Zn) samples. The samples were measured using an applied field of ±3 T with a PPMS-VSM.

The hysteresis curves (mass magnetization *M* *versus* apparent field *H*) of the four synthesized WHFs are given in Fig. 7 ▸. From the magnetic measurements it is seen that SrCo_2_Fe_16_O_27_ exhibits the highest *M*_s_ of 78.5 A m^2^ kg^−1^, while none of the samples show any appreciable coercivity or energy product (between 5–35 kA m^−1^ and 0.5–2.2 kJ m^−3^). This is due to the large crystallites splitting into multiple magnetic domains, which is consistent with the observed difference in broadening of the magnetic and nuclear diffraction signals. The result of multiple domains is a negligible coercivity, as domain wall motion allows continuous rotation of the magnetic moments rather than a collective flip as for single domain particles. Future reduction in the size of large particles may result in appreciable coercivities, as Ba*Me*_2_Fe_16_O_27_ has previously been demonstrated to have respectable anisotropy constants and coercivity (Lotgering *et al.*, 1961 ▸, 1980 ▸ *a*; Pullar, 2012 ▸). Inspecting the approach to magnetic saturation of the different samples in Fig. 7 ▸, it is clear SrCo_2_Fe_16_O_27_ varies from the three other WHFs in the curvature of magnetization. Considering the planar ordering seen in the neutron powder diffraction data, the difference could be explained by an easier reorientation of the magnetic moment with the *ab* plane rather than forcing the moment away for the uniaxial easy axis in the systems.

The macroscopically measured *M*_s,VSM_ of the four WHFs were compared with the calculated *M*_s,NPD_ from the refined magnetic moments of the NPD data (see Fig. 4 ▸ for numerical values). Across the four samples, *M*_s_ is higher for the VSM data compared with the NPD data. Notably, numerical equivalence between the calculated and measured magnetizations will most likely differ, given the distinct macroscopic and atomic nature of the two probes, as well as possibly unaccounted effects in the NPD refinement. Nevertheless, the trend from the magnetic measurements (*M*_s_ of SrCo_2_Fe_16_O_27_ > SrZn_2_Fe_16_O_27_ > SrNi_2_Fe_16_O_27_ > SrMg_2_Fe_16_O_27_) is consistent with the refined data. When accounting for the *M*_s_ of the impurity phases, SrZn_2_Fe_16_O_27_ has a higher *M*_s_ compared with SrCo_2_Fe_16_O_27_, but there are uncertainties of their *M*_s,NPD_ overlap. A comparison of *M*_s_ accounting for impurities and *M*_s_ values from the literature is given in Section S8 and Table S10 of the supporting information. Refinements where all seven crystallographic sites were constrained to have the same magnetic moment give rise to an increased calculated *M*_s_ at the cost of a significantly worse fit of the NPD data. The resulting *R*_mag_ and *R*_wp_ values for the two options are given in Table S6; μ_B_ and *M*_s_ are also given for the constrained option and compared with the individual and VSM values in Table S9. Looking at both the site occupation of the different *Me*s and the moment of the seven crystallographic sites, the underlying reason for the difference in *M*_s_ can be rationalized. The higher *M*_s_ derived from the NPD of SrCo_2_Fe_16_O_27_ in comparison with that of SrZn_2_Fe_16_O_27_ and SrNi_2_Fe_16_O_27_ can be explained by comparing the magnitude of the magnetic moments on the 12*k*_oct(R-S)_ site. This is higher for SrCo_2_Fe_16_O_27_ than for SrZn_2_Fe_16_O_27_, while the moment on the antiparallel sites 4*e*_tet(S)_, 4*f*_tet(S)_ and 4*f*_oct(R)_ are lower for SrCo_2_Fe_16_O_27_ than for SrNi_2_Fe_16_O_27_. When comparing SrNi_2_Fe_16_O_27_ with SrZn_2_Fe_16_O_27_, the former has a higher moment on 12*k*_oct(R-S)_ and the antiparallel sites also have significantly higher magnetic moments, especially 4*e*_tet(S)_ due to the Zn^2+^ contents on this site. SrMg_2_Fe_16_O_27_ exhibits the lowest *M*_s_, which is clearly understood from the low moment on 6*g*_oct(S-S)_ resulting from the high Mg^2+^ occupation on this site.

Further enhancement of W-type hexaferrite magnetic properties will be sought by means of nanostructuring, moving both to the single-domain region and optimizing the texture in a compacted magnet and ternary doping, with the aim of increasing *M*_s_ further while retaining sufficient *H*_c_ for optimal *BH*_max_.

## 4. Conclusions   

To improve the performance of magnetic materials and multiferroics, an in-depth understanding of their atomic structure is essential as it reveals the intrinsic magnetic properties of the compound. In W-type hexaferrites, the co-existence of divalent and trivalent metal ions results in a complex crystal structure allowing for a tunable magnetic structure. Based on combined refinement of neutron and X-ray powder diffraction data on sol-gel synthesized W-type hexaferrites (Sr*Me*_2_Fe_16_O_27_, *Me* = Mg, Co, Ni, Zn), we extracted the magnetic structure, where Mg, Ni and Zn exhibit uniaxial magnetic ordering along the crystallographic *c* axis, whereas SrCo_2_Fe_16_O_27_ is ordered magnetically in the *ab* plane.

The specific site occupation of the *Me* atoms was extracted from the joint refinements. Co and Ni have similar substituted structures with the majority of the transition metal sitting on the 6*g*_oct_ site, whereas Zn was mostly found on the 4*e*_tet_ site as opposed to Mg which it located on 6*g*_oct_. The atomic structure occupancies allow us to rationalize the Curie temperature where SrNi_2_Fe_16_O_27_, *T*_C_ = 500 (2)°C > SrCo_2_Fe_16_O_27_, *T*_C_ = 470 (3)°C > SrMg_2_Fe_16_O_27_, *T*_C_ = 435 (2)°C > SrZn_2_Fe_16_O_27_, *T*_C_ = 345 (3)°C. The affinity of the different cations for different crystallographic sites leads to an alteration of the magnetic moments resulting in variation in the strength of the superexchange interactions.

The *M*_s,VSM_ and the *M*_s,NPD_ calculated on the basis of the NPD reveal a simlar trend where the *M*_s_ is decreasing in the following order for the corrected VSM data: SrZn_2_Fe_16_O_27_, *M_s,VSM_* = 80.4 (3) > SrCo_2_Fe_16_O_27_, *M_s,VSM_* = 78.4 (2) > SrNi_2_Fe_16_O_27_, *M_s,VSM_* = 69.0 (3) > SrMg_2_Fe_16_O_27_, *M_s,VSM_* = 63.7 (2) A m^2^ kg^−1^. The relative variation of *M*_s_ between the different WHFs can be understood by the refined occupation fractions and magnetic moments on each sample.

Overall, the results illustrate the strength of combined synchrotron and neutron powder diffraction and the possibilities for rationalizing macroscopic properties from crystallographic and magnetic atomic structures. In conclusion, it is possible to tune magnetic properties and magnetic ordering of W-type hexaferrites by substitution of different divalent metals within the structure. This provides a handle for optimizing the magnetic properties of the compound, with the most promising results in terms of permanent magnets for SrCo_2_Fe_16_O_27_ and SrZn_2_Fe_16_O_27_.

## Supplementary Material

Supporting tables and figures. DOI: 10.1107/S2052252519003130/lt5018sup1.pdf

Zip of CIFs and structure factors. DOI: 10.1107/S2052252519003130/lt5018sup2.zip

Crystal structure: contains datablock(s) global, SrZn2Fe16O27, ZnFe2O4. DOI: 10.1107/S2052252519003130/lt5018sup3.cif

Crystal structure: contains datablock(s) global, SrZn2Fe16O27, ZnFe2O4. DOI: 10.1107/S2052252519003130/lt5018sup4.cif

Crystal structure: contains datablock(s) SrZn2Fe16O27. DOI: 10.1107/S2052252519003130/lt5018sup5.cif

Crystal structure: contains datablock(s) global, SrNi2Fe16O27, NiFe2O4. DOI: 10.1107/S2052252519003130/lt5018sup6.cif

Crystal structure: contains datablock(s) global, SrNi2Fe16O27, NiFe2O4. DOI: 10.1107/S2052252519003130/lt5018sup7.cif

Crystal structure: contains datablock(s) SrNi2Fe16O27. DOI: 10.1107/S2052252519003130/lt5018sup8.cif

Crystal structure: contains datablock(s) global, SrMg2Fe16O27, Sr2Mg2Fe28O46, MgFe2O4. DOI: 10.1107/S2052252519003130/lt5018sup9.cif

Crystal structure: contains datablock(s) global, SrMg2Fe16O27, Sr2Mg2Fe28O46, MgFe2O4. DOI: 10.1107/S2052252519003130/lt5018sup10.cif

Crystal structure: contains datablock(s) SrMg2Fe16O27. DOI: 10.1107/S2052252519003130/lt5018sup11.cif

Crystal structure: contains datablock(s) global, SrCo2Fe16O27, CoFe2O4. DOI: 10.1107/S2052252519003130/lt5018sup12.cif

Crystal structure: contains datablock(s) global, SrCo2Fe16O27, CoFe2O4. DOI: 10.1107/S2052252519003130/lt5018sup13.cif

Crystal structure: contains datablock(s) SrCo2Fe16O27. DOI: 10.1107/S2052252519003130/lt5018sup14.cif

CCDC references: 1912938, 1912939, 1913135, 1913136, 1913174, 1913175, 1913179, 1913180, 1913181
