# Supplementary material for: Structure and magnetic properties of W-type hexaferrites
Source: IUCrJ. 2019 Apr 30;6(Pt 3):492–9. doi: 10.1107/S2052252519003130 (PMC6503931; doi:10.1107/S2052252519003130)
Supplement: Supplementary file 1 [file m-06-00492-sup1.pdf]

IUCrJ

Volume 6 (2019)

Supporting information for article:

Structure and Magnetic Properties of W-type Hexaferrites

Mathias I. Mørch, Jakob V. Ahlburg, Matilde Saura-Múzquiz, Anna Z. Eikeland and Mogens Christensen

S1. Combined refinements of WHFs

In the following figures the result of the combined refinements is shown for W-Type Hexaferrites (WHFs)

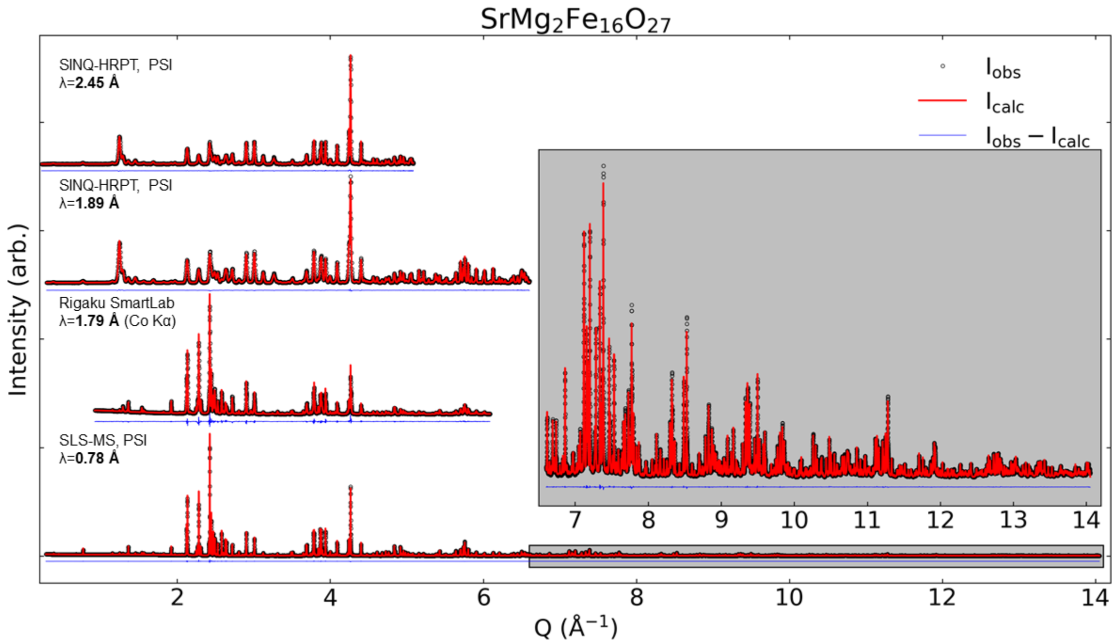

Figure S1 Combined refinement from the four indicated sources for $SrMg_2Fe_{16}O_{27}$. Dots are the observed intensities, the red line the calculated intensities for the model, and the blue the difference between the model and the observed.

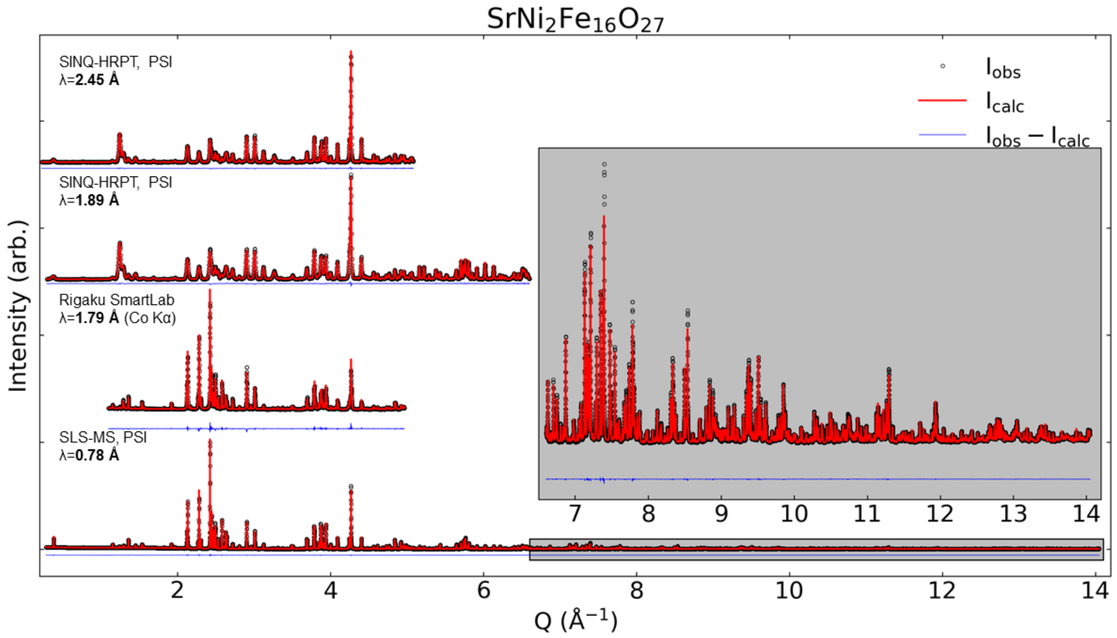

Figure S2 Combined refinement for the four indicated sources for $SrNi_2Fe_{16}O_{27}$. Dots are the observed intensities, the red line the calculated intensities for the model, and the blue the difference between the model and the observed.

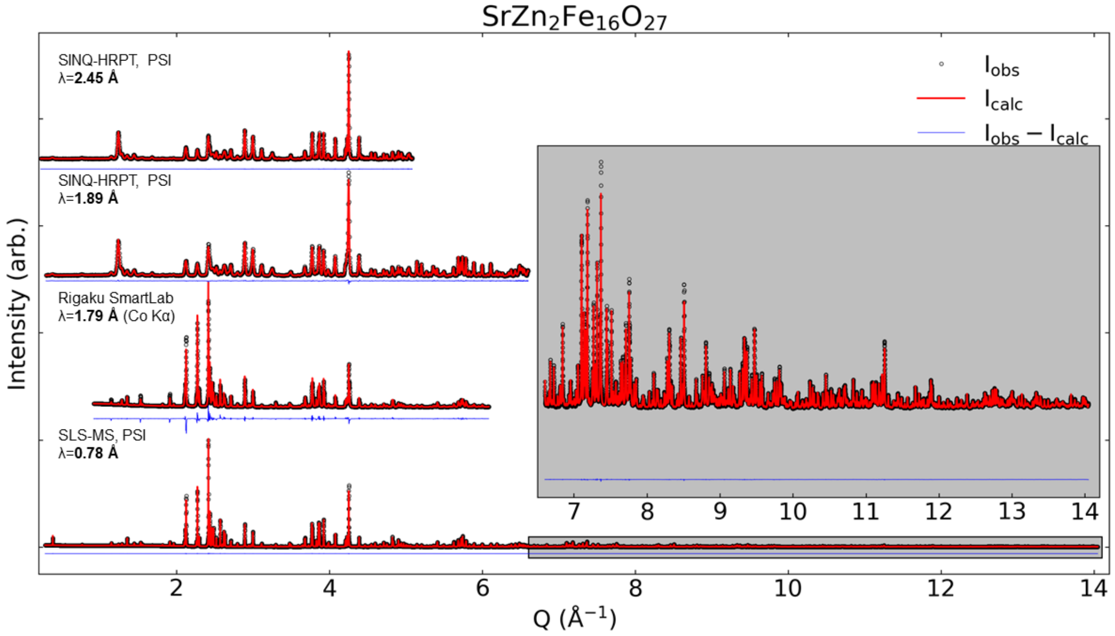

Figure S3 Combined refinement for the four indicated sources for $SrZn_2Fe_{16}O_{27}$. Dots are the observed intensities, the red line the calculated intensities for the model, and the blue the difference between the model and the observed.

S2. Curie Temperature measurements

The T_C was determined by thermogravimetry as described in the main text. The temperature is determined by the intercept of a line fitted to the mass increase near T_C and a line fitted to the signal of the mass before.

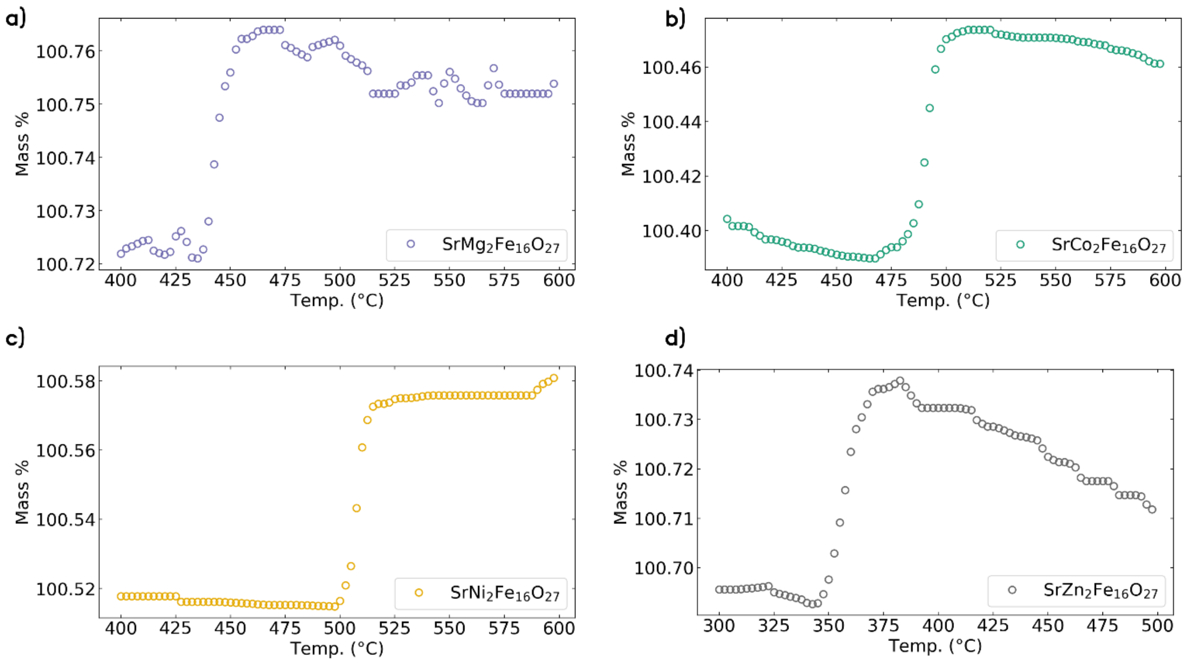

Figure S4 Thermogravimetric measurements of the Curie Temperature for a) $SrMg_2Fe_{16}O_{27}$, b) $SrCo_2Fe_{16}O_{27}$, c) $SrNi_2Fe_{16}O_{27}$ and d) $SrZn_2Fe_{16}O_{27}$ shown as mass % vs Temperature in °C for the range of interest.

S3. Comparison of occupation to literature

In the following 3 tables *Me* occupation is shown and compared to literature.

Table S1 Comparison between Co occupation between this work for $SrCo_2Fe_{16}O_{27}$ and (Collomb, Wolfers, *et al.*, 1986) $BaCo_2Fe_{16}O_{27}$ for both Powder (p) and Single-Crystal (sc) data.

$SrCo_2Fe_{16}O_{27}$ and $BaCo_2Fe_{16}O_{27}$

	Site	$SrCo_2Fe_{16}O_{27}$	$BaCo_2Fe_{16}O_{27}$(p)	$BaCo_2Fe_{16}O_{27}$(sc)
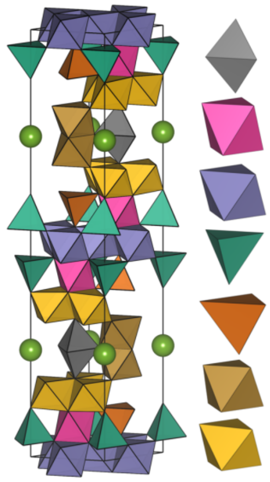	4f ½bi(R)	0	0	0
	4f oct(S)	0.078(15)	0.096(9)	0.120(9)
	6g oct(S-S)	0.333(11)	0.441(7)	0.463(8)
	4e tet(S)	0.140(14)	0.108(9)	0.084(9)
	4f tet(S)	0.111(16)	0.067(6)	0.043(9)
	4f oct(R)	0.017(15)	0	0
	12k oct(R-S)	0.079(10)	0	0
	Total	2.17(18)	1.87(6)	1.88(7)

Table S2 Comparison between Mg occupation between this work $SrMg_2Fe_{16}O_{27}$ and (Collomb, Abdelkader, *et al.*, 1986) $BaMg_2Fe_{16}O_{27}$ for both Powder (p) and single-crystal (sc)

$SrMg_2Fe_{16}O_{27}$ and $BaMg_2Fe_{16}O_{27}$

Structure	Site	$SrMg_2Fe_{16}O_{27}$	$BaMg_2Fe_{16}O_{27}$ (p)	$BaMg_2Fe_{16}O_{27}$ (sc)
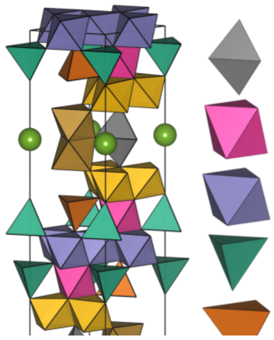	4f ½bi(R)	0	0.00(2)	0.0082(17)
	4f oct(S)	0.099(3)	0.086(7)	0.120(9
	6g oct(S-S)	0.458(2)	0.405(4)	0.378(8)
	4e tet(S)	0.035(3)	0.148(6)	0.160(10)
	4f tet(S)	0.024(3)	0.124(7)	0.101(9)

$4f_{\mathrm{oct(R)}}$	0	0	0
$12k_{\mathrm{oct(R\text{-}S)}}$	0	0	0
Total	1.68(2)	1.93(4)	1.98(10)

Table S3 Comparison of Zn occupation in $SrZn_2Fe_{16}O_{27}$ between this work and (Graetsch *et al.*, 1986) single crystal (sc).

Structure	Site	$SrZn_2Fe_{16}O_{27}$	$SrZn_2Fe_{16}O_{27}$ (sc)
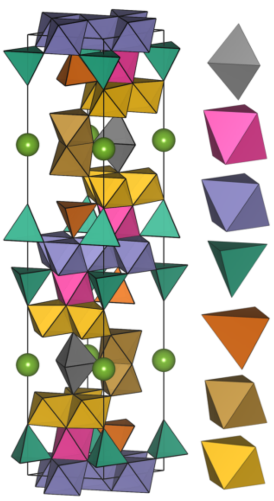	$4f_{\mathrm{½bi(R)}}$	0	0
	$4f_{\mathrm{oct(S)}}$	0.028(9)	0
	$6g_{\mathrm{oct(S\text{-}S)}}$	0	0
	$4e_{\mathrm{tet(S)}}$	0.551(8)	0.75
	$4f_{\mathrm{tet(S)}}$	0.236(9)	0.25
	$4f_{\mathrm{oct(R)}}$	0.033(8)	0
	$12k_{\mathrm{oct(R\text{-}S)}}$	0.035(5)	0
	Total	1.90(5)	2

Table S4 Refined unit cell parameters and isotropic thermal parameters.

	$SrMg_2Fe_{16}O_{27}$	$SrCo_2Fe_{16}O_{27}$	$SrNi_2Fe_{16}O_{27}$	$SrZn_2Fe_{16}O_{27}$
$a = b$ [Å]	5.8955(1)	5.8946(1)	5.8877(3)	5.90922(3)
c [Å]	32.7308(6)	32.7411(7)	32.702(2)	32.8293(2)
B_{iso}(Sr) [Å^2]	0.92(1)	0.86(1)	0.68(1)	0.93(1)
B_{iso}(Fe/*Me*) [Å^2]	0.262(2)	0.242(2)	0.070(2)	0.272(2)
B_{iso}(O) [Å^2]	0.371(9)	0.368(8)	0.141(9)	0.379(9)

S4. Highlighted magnetic signal

In the following figures the magnetic signal is highlighted for each of the 4 samples and in the end compared to each other. As the propagation vector **k** = 0, the magnetic and nuclear unit cell coincide and only one set of Bragg peaks is shown per phase. Shown data is from SINQ-HRPT at PSI with

chosen wavelength 2.45 Å. Grey dots are observed data, the grey line is the calculated pattern, and the blue line is the difference. The red line is the magnetic contribution to the calculated pattern and the vertical bars indicate the given phases (see legend in figure)

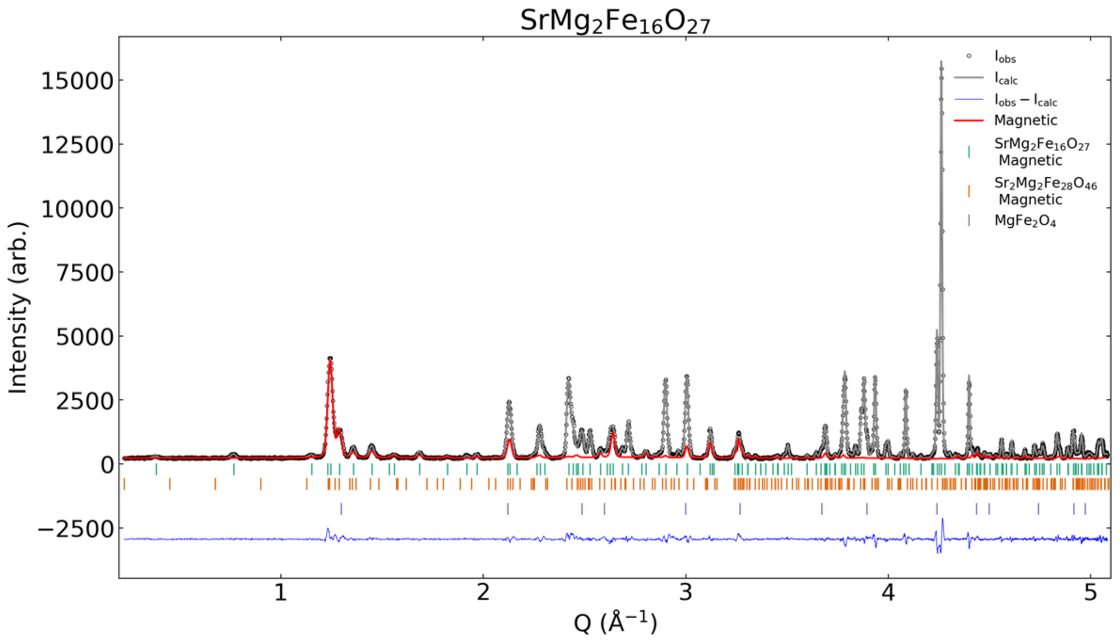

Figure S5 Highlighted magnetic contribution to the pattern for $SrMg_2Fe_{16}O_{27}$.

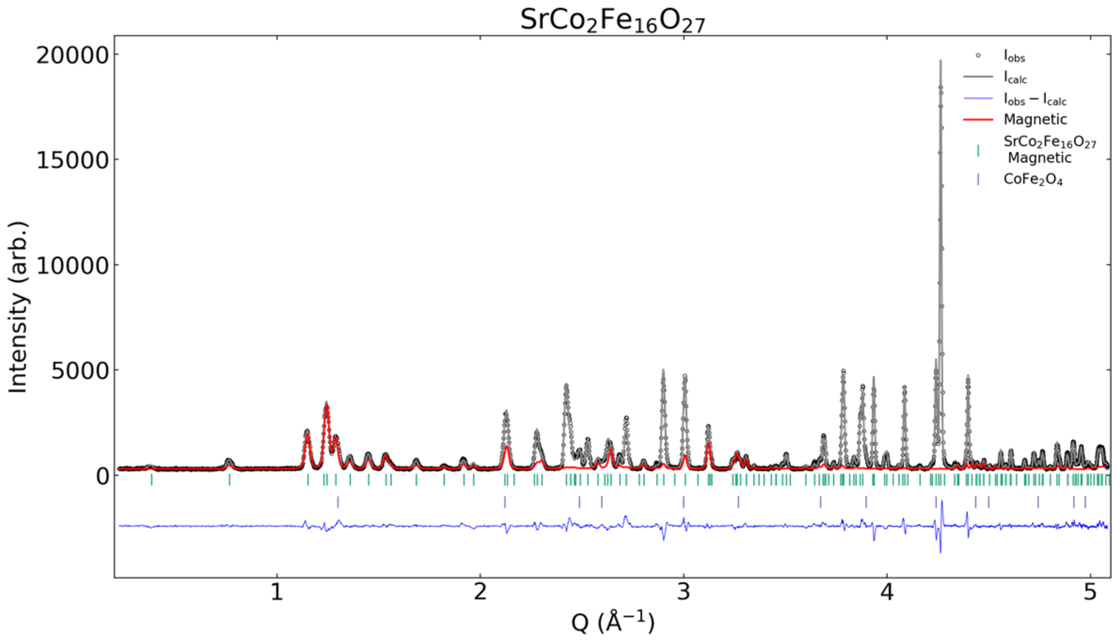

Figure S6 Highlighted magnetic contribution to the pattern for $SrCo_2Fe_{16}O_{27}$

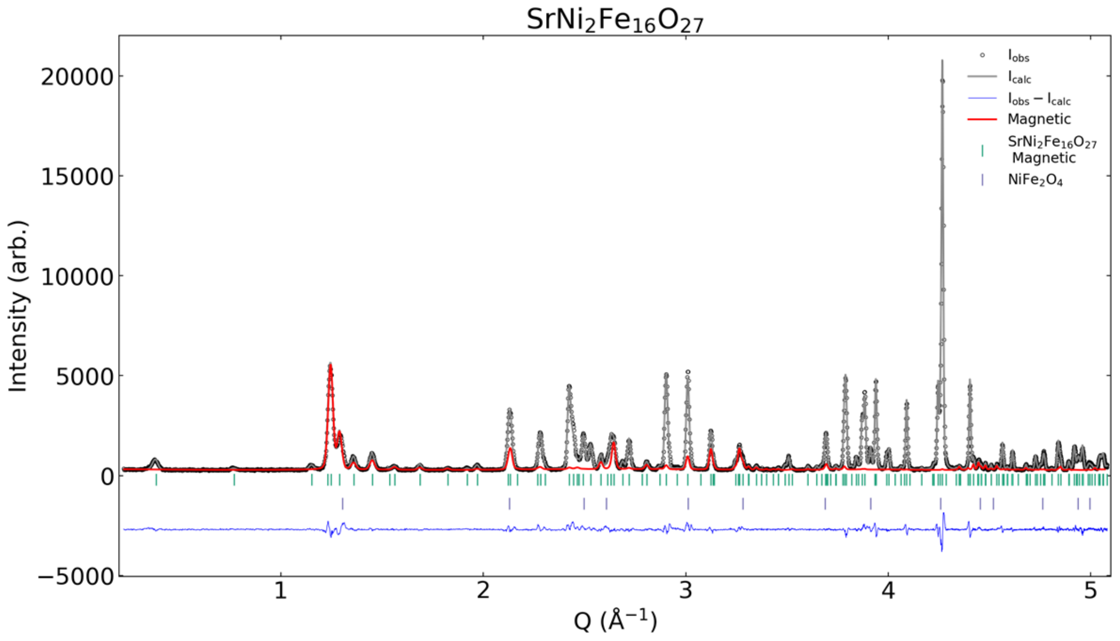

Figure S7 Highlighted magnetic contribution to the pattern for $SrNi_2Fe_{16}O_{27}$

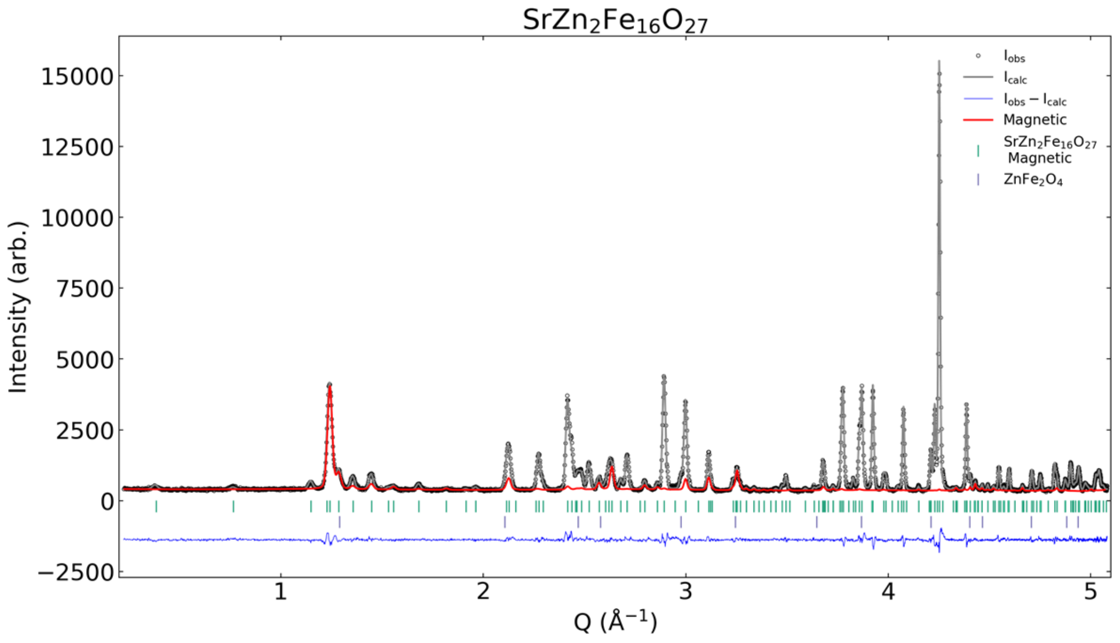

Figure S8 Highlighted magnetic contribution to the pattern for $SrZn_2Fe_{16}O_{27}$

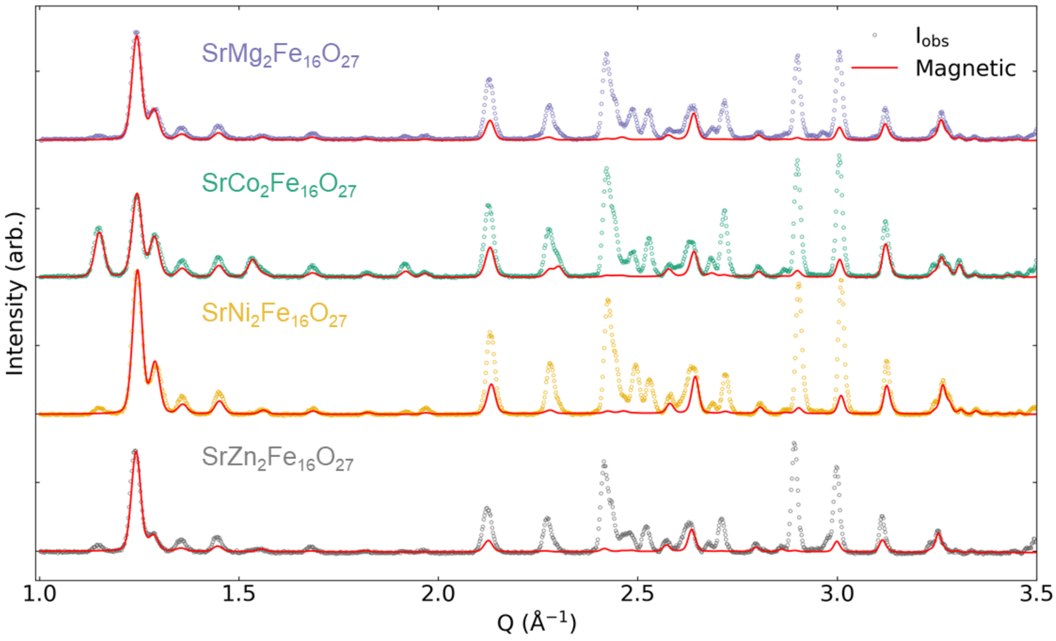

Figure S9 Comparison of the magnetic contribution to the neutron powder diffraction pattern. Top

S5. Determination of Magnetic symmetry

The chosen Shubnikov group was found by consulting the Magnetic Group Tables (Litvin, 2013) and analyzing the symmetry with the following two assumptions: 1) The nuclear symmetry is kept $P6_3/mmc$ and only magnetic groups within $P6_3/mmc$ were considered. 2) Only symmetries with ferromagnetic ordering on individual Wyckoff sites occupied by magnetic ions (4f, 4e, 6g and 12k) were considered as the material showed ferrimagnetic hysteresis. From this the Shubnikov group $P6_3/mm'c'$ (194.8.1501) emerged as the only candidate fulfilling these requirements and a net moment along the c-axis is allowed on all four Wyckoff sites.

S6. Refined atomic coordinates

In the following table the refined atomic coordinates are shown for all four samples.

Table S5 Atomic coordinates for the four samples.

Site	$SrMg_2Fe_{16}O_{27}$			$SrCo_2Fe_{16}O_{27}$			$SrNi_2Fe_{16}O_{27}$			$SrZn_2Fe_{16}O_{27}$		
	x	y	z	x	y	z	x	y	z	x	y	z
$4f_{½bi(R)}$	1/3	2/3	0.74553(4)	1/3	2/3	0.74568(4)	1/3	2/3	0.74559(4)	1/3	2/3	0.74524(4)
$4f_{oct(S)}$	1/3	2/3	-0.07440(2)	1/3	2/3	-0.07422(2)	1/3	2/3	-0.07396(2)	1/3	2/3	-0.07528(2)
$6g_{oct(S-S)}$	1/2	0	0	1/2	0	0	1/2	0	0	1/2	0	0
$4e_{tet(S)}$	0	0	0.05569(2)	0	0	0.05608(2)	0	0	0.05545(2)	0	0	0.05650(2)
$4f_{tet(S)}$	1/3	2/3	0.09273(2)	1/3	2/3	0.09304(2)	1/3	2/3	0.09273(2)	1/3	2/3	0.09400(2)
$4f_{oct(R)}$	1/3	2/3	0.20831(2)	1/3	2/3	0.20840(2)	1/3	2/3	0.20826(2)	1/3	2/3	0.20856(2)
$12k_{oct(R-S)}$	0.83561(9)	0.67119(18)	0.15106(1)	0.83536(9)	0.67068(17)	0.15114(1)	0.83534(9)	0.67064(17)	0.15083(1)	0.83606(9)	0.67214(19)	0.15139(1)
12k_O1	0.82479(31)	0.64970(79)	0.03633(5)	0.82515(35)	0.65040(71)	0.03663(5)	0.82482(37)	0.64968(73)	0.03594(5)	0.82234(39)	0.64474(78)	0.03516(6)
4f_O2	1/3	2/3	0.03394(9)	1/3	2/3	0.03495(9)	1/3	2/3	0.03445(9)	1/3	2/3	0.0339(1)
12k_O3	0.51156(39)	0.02304(77)	0.11083(5)	0.51082(36)	0.02152(73)	0.11088(5)	0.51075(38)	0.02142(75)	0.11070(5)	0.51226(41)	0.02441(81)	0.11054(5)
4e_O4	0	0	0.11318(9	0	0	0.11353(8)	0	0	0.11275(8)	0	0	
12k_O5	0.16204(43)	0.32414(86)	0.18008(5)	0.16317(39)	0.32632(79)	0.18000(5)	0.16251(41)	0.32500(82)	0.18006(5)	0.16169(5)	0.3235(1)	0.18037(15)
4f_O6	1/3	2/3	-0.18163(9)	1/3	2/3	-0.18080(9)	1/3	2/3	-0.18130(9)	1/3	2/3	-0.18055(9)
6h_O7	0.48388(52)	0.9678(10)	1/4	0.48320(48)	0.96651(96)	1/4	0.48370(49)	0.96740(99)	1/4	0.48412(54)	0.9683(11)	1/4

S7. Broadening parameters and R-Values

In the following tables a comparison of different refinement models is given. Figure S11and Figure S12 show the Pseudo Voight FWHM of the different instruments. Table S5 shows final R values for the four samples. Table S6 shows a comparison of R_{wp} for two models. One is the broadening parameter Y being locked to be equal for both the magnetic contribution and the nuclear. While the second allows them to have independent values. Table S7 shows a comparison of two refinement models for the magnetic moment on each crystallographic site. One constrains all moments to be of equal magnitude, while the other is individual magnitude.

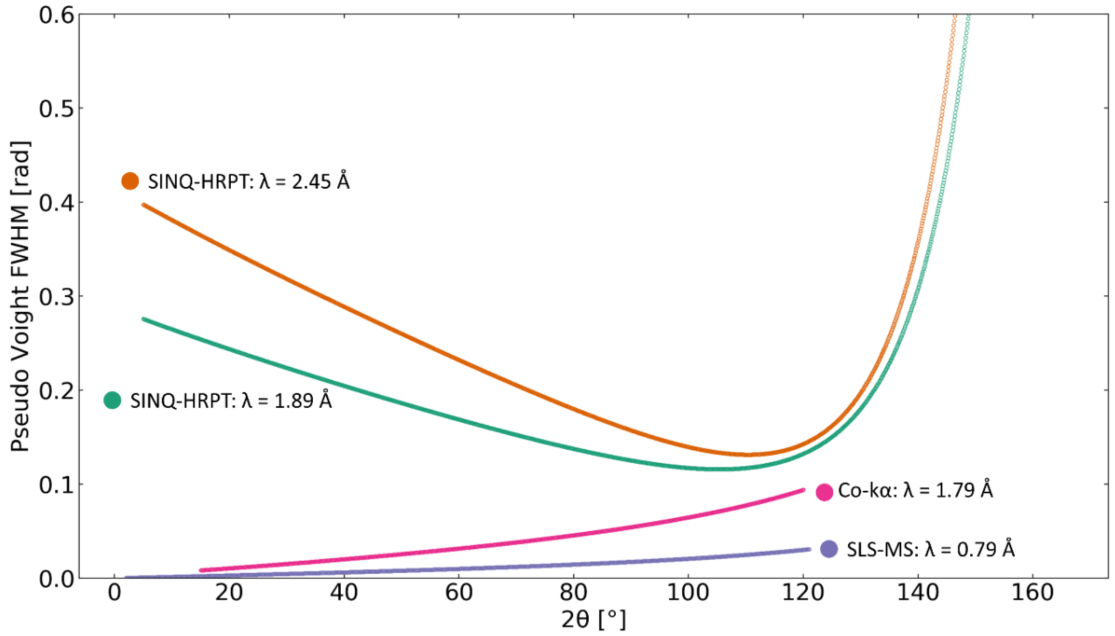

Figure S10 Pseudo Voight FWHM [rad] vs 2θ [°]

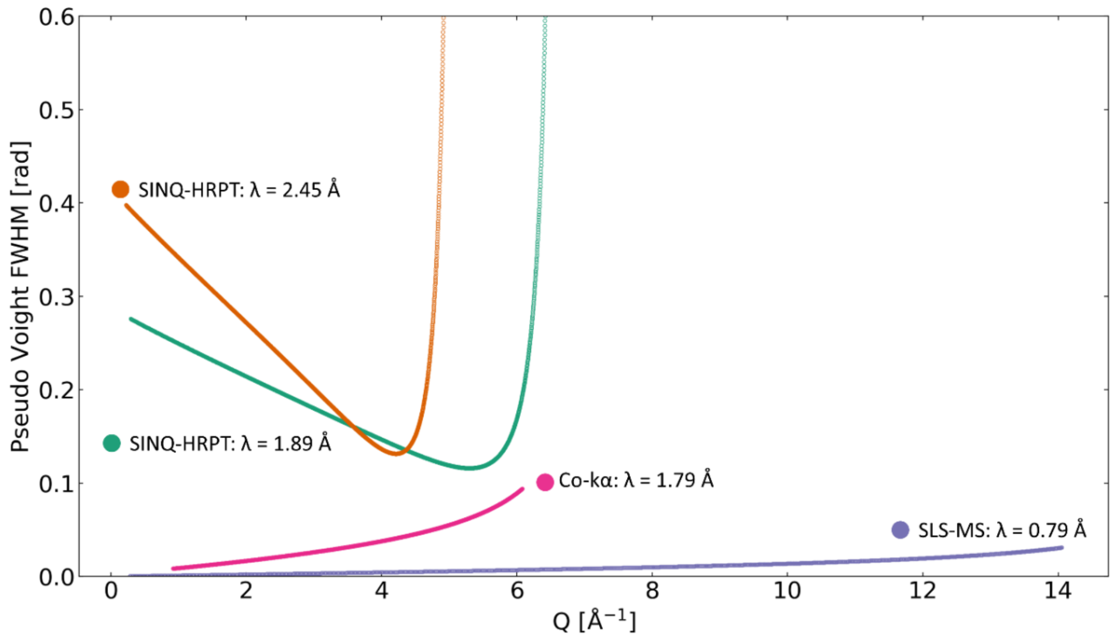

Figure S11 Pseudo Voight FWHM [rad] vs Q [Å^{-1}]

Table S6 Final R-Values for the refined patterns

	R_{Bragg} / $R_{Magnetic}$ / R_{wp} SINQ-HRPT λ = 1.89	R_{Bragg} / $R_{Magnetic}$ /R_{wp} SINQ-HRPT λ = 2.45	R_{Bragg} / R_{wp} Co-source λ = 1.79	R_{Bragg} / R_{wp} SLS-MS λ = 0.78
$SrMg_2Fe_{16}O_{27}$	2.68 / 2.69 / 5.28	2.57 / 4.90 / 7.50	4.95 / 8.26	4.83 / 4.45
$SrCo_2Fe_{16}O_{27}$	4.28 / 5.32 / 6.33	4.69 / 6.84 / 8.57	9.47 / 14.50	3.97 / 3.52
$SrNi_2Fe_{16}O_{27}$	2.64 / 2.45 / 5.46	2.74 / 3.02 / 7.13	5.71 / 10.60	4.17 / 4.19
$SrZn_2Fe_{16}O_{27}$	2.83 / 2.17 / 4.43	3.12 / 2.70 / 6.11	10.80 / 13.00	4.21 / 3.94

Table S7 Comparison of Broadening Parameters for data from λ = 2.45 Å, SINQ-HRPT at PSI

Sample	Y_{locked}	$R_{wp, locked}$	Y_{mag}	Y_{nuc}	R_{wp}
$SrMg_2Fe_{16}O_{27}$	0.007743	5.66	0.082461	0.007961	5.28
$SrCo_2Fe_{16}O_{27}$	0.008154	6.59	0.074360	0.008966	6.33
$SrNi_2Fe_{16}O_{27}$	0.006572	5.83	0.081877	0.007130	5.46
$SrZn_2Fe_{16}O_{27}$	0.010501	4.65	0.073453	0.011324	4.43

Table S8 Comparison of R-factors (Neutron λ = 2.45) for equal and individual moments on crystallographic sites

(R_{Mag} / R_{wp})	$SrMg_2Fe_{16}O_{27}$	$SrCo_2Fe_{16}O_{27}$	$SrNi_2Fe_{16}O_{27}$	$SrZn_2Fe_{16}O_{27}$
Equal	7.45 / 7.82	8.12 / 8.84	6.73 / 7.63	8.29 / 6.71
Individual	4.90 / 7.50	6.84 / 8.57	3.02 / 7.13	2.70 / 6.11

Table S9 Magnitude of the average moment and the M_s derived from this for equal and individual moments. M_s also shown for VSM data.

	$SrMg_2Fe_{16}O_{27}$	$SrCo_2Fe_{16}O_{27}$	$SrNi_2Fe_{16}O_{27}$	$SrZn_2Fe_{16}O_{27}$
Avg. Moment (μ_B)	4.41(6)	3.35(3)	4.03(7)	3.30(7)
M_s (Am^2/kg) Equal	93.6(3)	73.3(1)	88.2(3)	71.7(2)
M_s (Am^2/kg) Individual	41(3)	59(3)	50(5)	57(5)
M_s (Am^2/kg) VSM	60.3(1)	78.5(1)	67.7(1)	76.2(1)

S8. M_S comparison

The secondary phases have the following saturation magnetizations, where for spinel-ferrites the calcined/ceramic synthetized samples are chosen: $BaMg_2Fe_{28}O_{46}$(X-Type Hexaferrite): 63.29 Am^2/kg (Kagdi et al., 2018), $MgFe_2O_4$: 25 Am^2/kg (Kulkarni & Joshi, 1986), $CoFe_2O_4$:80 Am^2/kg (Berkowitz et al., 1980), $NiFe_2O_4$: 50 Am^2/kg (Berkowitz et al., 1980) and $ZnFe_2O_4$: 1 Am^2/kg (Jeyadevan et al., 1994)

The unit cells of the respective pure spinel-ferrites are in good agreement with litterature justifying calculating the corrected saturation magnetization with their M_s from literature. The saturation magnetization of the four WHFs is calculated from the wt% of the respective phases from the refinement.

$$M_{s_{WHF_{Calc}}} = \frac{M_{s_{measured}} - M_{s_{secondaryphase,litterature}} \cdot wt\%_{secondaryphase}}{wt\%_{WHF}}$$

Table S10 Comparison of M_S from measured with VSM and accounting for impurity phases.

	$SrMg_2Fe_{16}O_{27}$	$SrCo_2Fe_{16}O_{27}$	$SrNi_2Fe_{16}O_{27}$	$SrZn_2Fe_{16}O_{27}$
M_s Measured Am^2/kg	60.3(1)	78.5(1)	67.7(1)	76.2(1)
M_s Calculated Am^2/kg	63.7(2)	78.4(2)	69.0(3)	80.4(3)

S9. Undescribed peaks in $SrMg_2Fe_{16}O_{27}$

In the subsequent figures undescribed peaks seen only in the high-quality synchrotron data from SLS-MS of $SrMg_2Fe_{16}O_{27}$ is shown.

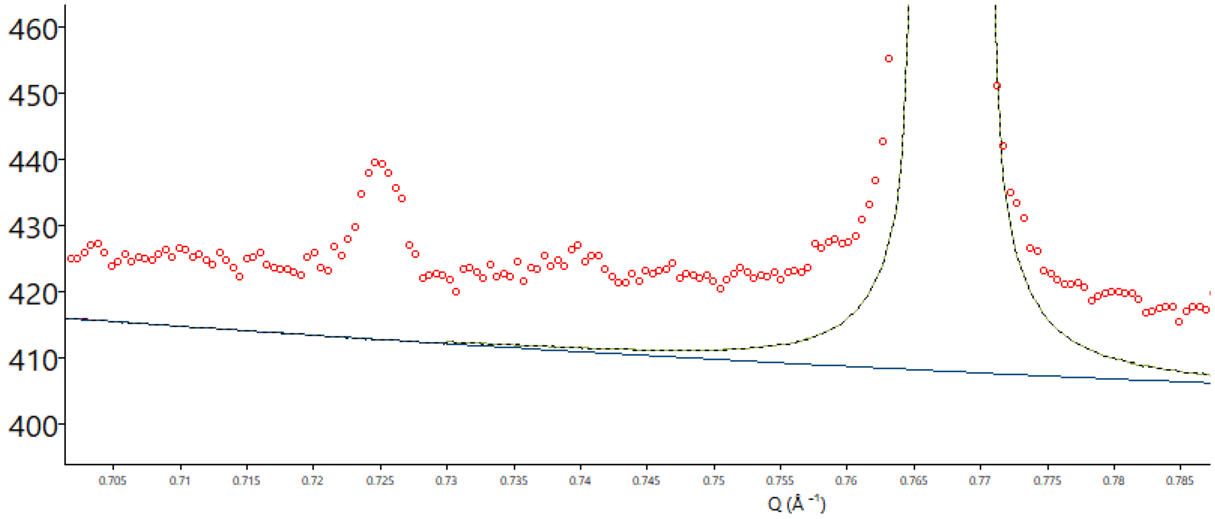

Figure S12 Undescribed peak at 0.72 $Å^{-1}$ seen in SLS-MS data.

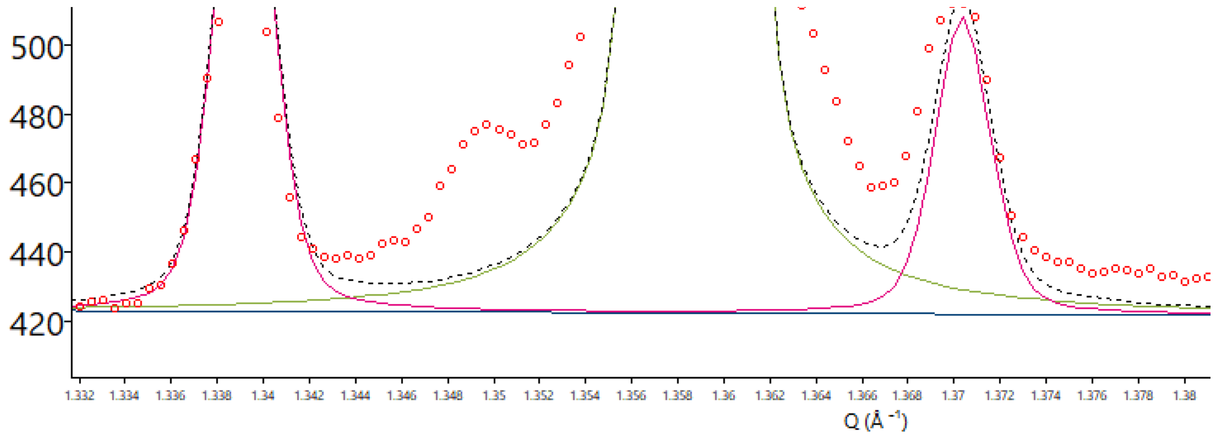

Figure S13 Undescribed peak at 1.35 $Å^{-1}$ seen in SLS-MS data.

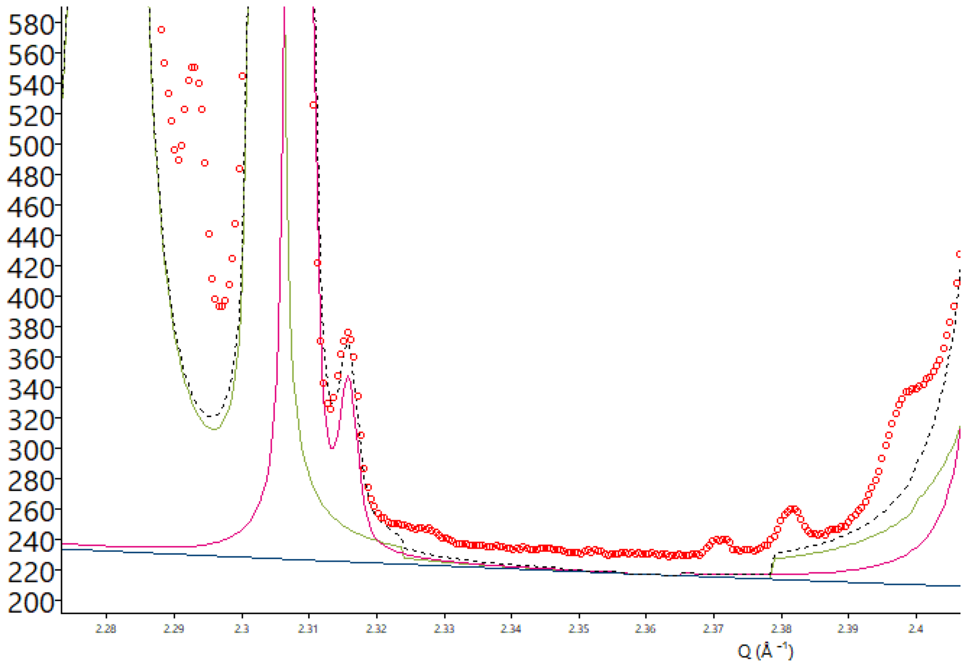

Figure S14 Undescribed peaks at 2.295, 2.37, 2.38 and 2.395 Å^{-1} seen in SLS-MS data.

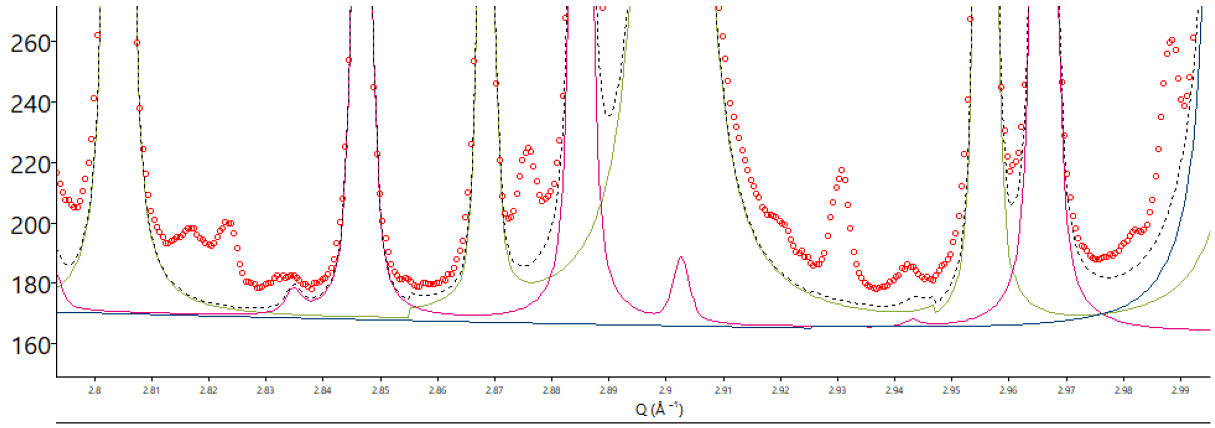

Figure S15 Undescribed peaks at 2.82, 2.875, 2.93 and 2.985 Å^{-1} seen in SLS-MS data.

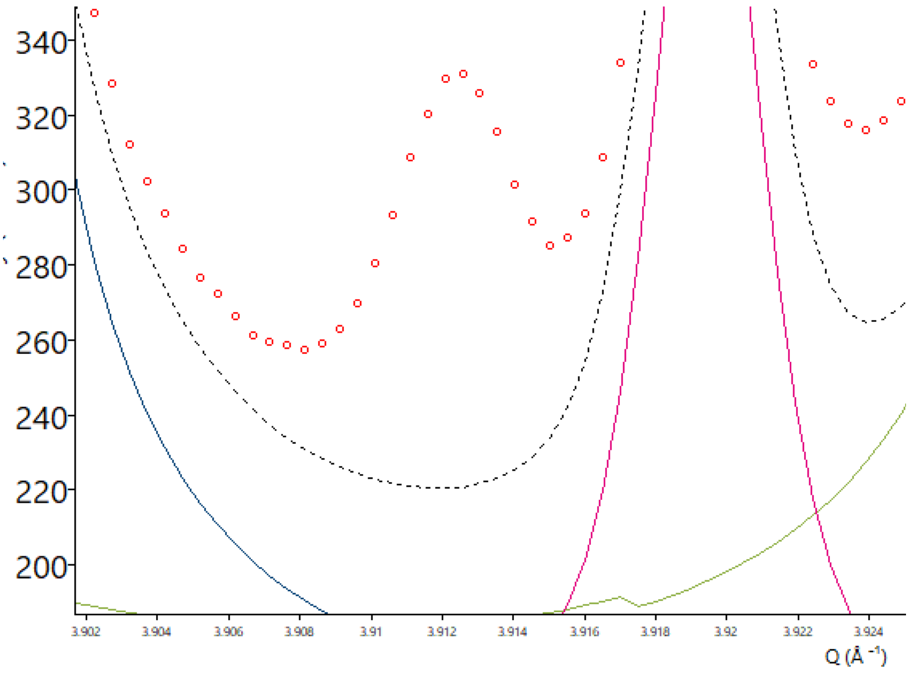

Figure S16 Undescribed peak at 3.912 Å^{-1} seen in SLS-MS data.
